# Hyperchloremia is not associated with AKI or death in septic shock patients: results of a post hoc analysis of the “HYPER2S” trial

**DOI:** 10.1186/s13613-019-0570-3

**Published:** 2019-08-22

**Authors:** Morgane Commereuc, Camille Nevoret, Peter Radermacher, Sandrine Katsahian, Pierre Asfar, Frédérique Schortgen, HYPER2S investigators

**Affiliations:** 1 Service de Réanimation et Surveillance Continue Adulte, Centre hospitalier intercommunal de Créteil, 94000 Créteil, France; 2 INSERM, UMR_S 1138, Université Paris Descartes, Sorbonne Universités, UPMC Université Paris 06, Centre de Recherche des Cordeliers, Paris, France; 3 Unité d’Épidémiologie et de Recherche Clinique, Assistance Publique - Hôpitaux de Paris, Hôpital Européen Georges-Pompidou, Paris, France; 4 INSERM, Centre d’Investigation Clinique 1418, Module Épidémiologie Clinique, Paris, France; 5 Institut für Anästhesiologische Pathophysiologie und Verfahrensentwicklung, Universitätsklinikum Ulm, Helmholtzstr 8-1, 89081 Ulm, Germany; 6 Département de Médecine Intensive-Réanimation et Médecine Hyperbare, CHU d’Angers, Angers, France; 7 INSERM U955 Equipe 13, Faculté de Médecine, 94010 Créteil, France

**Keywords:** Crystalloids, Hyperchloremia, Acute kidney injury, Hyperlactatemia, Metabolic acidosis, Septic shock

## Abstract

**Background:**

Recent data suggest that hyperchloremia induced by fluid resuscitation is associated with acute kidney injury (AKI) and mortality, particularly in sepsis. Experimental studies showed that hyperchloremia could affect organ functions. In patients with septic shock, we examined the relationship between serum chloride concentration and both renal function and survival.

**Methods:**

Post hoc analysis of the “HYPER2S” trial database (NCT01722422) including 434 patients with septic shock randomly assigned for resuscitation with 0.9% or 3% saline. Metabolic parameters were recorded up to 72 h. Metabolic effects of hyperchloremia (> 110 mmol/L) were studied stratified for hyperlactatemia (> 2 mmol/L). Cox models were constructed to assess the association between chloride parameters, day-28 mortality and AKI.

**Results:**

413 patients were analysed. The presence of hyperlactatemia was significantly more frequent than hyperchloremia (62% versus 71% of patients, respectively, *p* = 0.006). Metabolic acidosis was significantly more frequent in patients with hyperchloremia, no matter the presence of hyperlactatemia, *p* < 0.001. Adjusted risk of AKI and mortality were not significantly associated with serum chloride, hyperchloremia, maximal chloremia and delta chloremia (maximal-H0 [Cl]).

**Conclusions:**

Despite more frequent metabolic acidosis, hyperchloremia was not associated with an increased risk for AKI or mortality.

*Trial registration* ClinicalTrials.gov, identifier: NCT01722422, registered 2 November 2012

## Background

Fluid therapy is commonly administered in patients with septic shock. Crystalloid solutions are recommended for the initial resuscitation of sepsis [1]. In the past decades, normal saline (0.9% NaCl), was the most frequently used fluid in adult ICU patients [2]. Normal saline, which contains 154 mmol/L of both sodium and chloride, can induce hyperchloremic acidosis with recently suggested adverse events [3]. Acute kidney injury (AKI) represents the most alarming one. Balanced crystalloid solutions, in which chloride anions are replaced by lactate or acetate, are increasingly used alternatives [2].

Three recent pragmatic RCTs have been conducted to compare the effectiveness of balanced crystalloids against normal saline for fluid therapy in heterogeneous populations of ICU patients [4–6]. None of them found a significant difference in AKI and mortality. However, in the “SMART” trial, normal saline significantly increased the incidence of major adverse kidney events (MAKE) within 30 days, a composite end point of persistent renal injury, renal replacement therapy and death [4]. Moreover, in the pre-specified subgroup of patients with sepsis, mortality was significantly higher in the saline group [4]. The significant reduction in mortality among patients with sepsis receiving balanced crystalloids has been confirmed in a recent meta-analysis [7]. However, no randomized study is available specifically designed to compare outcomes of septic patients resuscitated with balanced crystalloids or saline. In the absence of chloremia monitoring in the “SPLIT” trial [5] or a significant, but limited difference in chloremia in the SALT [6] and SMART trials [4], a cause-effect relationship between hyperchloremia and outcome remains uncertain. Moreover, patients included in these three RCTs were of low severity and only received a limited amount of fluids. The effects of chloride content and of hyperchloremia are, therefore, still controversial [3, 8]. Patients with septic shock requiring larger volumes of fluids and being at higher risk for AKI could be more exposed to the risk of chloride load. In this population, serum lactate represents an additional strong anions involved in acid–base balance. Compared to lactic acidosis, hyperchloremic acidosis has been experimentally shown to increase inflammation and hypotension [9]. Therefore, this post hoc analysis of the “HYPER2S” study comparing normal saline to 3% NaCl for septic shock resuscitation, we assessed the impact of hyperchloremia on AKI and mortality, stratified by lactatemia [10].

## Methods

For all participating centres, the “HYPER2S” trial was approved by the ethics committee of the Angers University Hospital. Written informed consent was obtained from all patients, their next of kin, or another surrogate decision-maker, as appropriate. The “HYPER2S” trial was registered with Clinicaltrial.gov (NCT 01722422).

### “HYPER2S” cohort

The present study is a post hoc analysis of the “HYPER2S” trial database, of which primary results were previously published [10]. Adult patients with septic shock admitted in the 22 participating ICUs were considered eligible for enrolment. Inclusion criteria comprised the need for epinephrine and/or norepinephrine infusion for less than 6 h at a minimum infusion rate of 0.1 µg/Kg/min, mechanical ventilation and plasma sodium concentration ≥ 130 mmol/L or ≤ 145 mmol/L. Patients were randomized to be treated with 0.9% (isotonic) saline or 3% (hypertonic) saline. Fluids were infused in a double-blind fashion during the first 72 h after inclusion. Study fluids were stopped if [Na^+^] > 155 mmol/L or > 12 mmol/L increase over 24 h together with a switch to open label isotonic saline. After the 72-h period of study fluid resuscitation, open label 0.9% saline was used for the remainder of the ICU stay in all patients.

Metabolic parameters and arterial blood gazes were concomitantly recorded at inclusion (H0), H12, H24 and H72. Serum creatinine and urine output (UO) were recorded daily up to D7 and the need for RRT up to day-28.

The trial was stopped prematurely for safety reasons after enrolment of 442 patients (434 analyzable), due to a toxic effect of hyperoxia. At study stop, day-28 mortality was 37% in the isotonic group vs 42% in the hypertonic saline group (*p* = 0.25)

*Definitions* The definitions of metabolic and acid-base disorders are provided in Additional file 1: Table S1. Hyperchloremia was defined by [Cl] ≥ 110 mmol/L. This cut off value has been shown to be associated with more AKI and mortality rate in previous studies [11–13]. Definitions of acidemia (pH < 7.35) and of metabolic acidosis (bicarbonate < 22 mmol/L) were based on previous studies as well [14]. Hyperlactatemia was defined by a serum concentration > 2 mmol/L according to the sepsis-3 definition [15].

Because cumulative urine output was recorded daily only, AKI definition was based on serum creatinine criteria [16]. Serum creatinine at inclusion (i.e. just before exposure to chloride rich crystalloids) was used for baseline value. The proportion of patients with Major Adverse Kidney Events (MAKE), a composite of death, new receipt of RRT or RRT dependence at day 28, was computed and censored at hospital discharge or 28 days after H0, whichever came first [4, 17].

### Data analysis

In this post hoc analysis, only patients with available data on chloremia were analysed. Patients were classified according to the presence of at least one recorded episode of hyperchloremia or not. Patients’ characteristics were compared at baseline (H0), metabolic evolution was compared up to H72 and outcomes up to day-28.

In the context of septic shock, hyperlactatemia is a determinant of acid-base balance and represents a strong marker of severity with a significant higher risk of death. We have previously confirmed in the “HYPER2S” cohort that hyperlactatemia is a marker of higher severity and has a strong impact on outcome [18]. Analysis of the role of hyperchloremia on acid-base balance and outcomes was, therefore, adjusted to serum lactate concentration. Quantitative variables were expressed as median and 25th–75th interquartile range (IQR) and were compared using the Mann–Whitney test. Qualitative variables were compared using χ2 or Fisher’s exact test. General mixed models were developed to compare the evolution of metabolic parameters and renal function over time. The model was linear for pH and non-linear for chloride, lactate, bicarbonate, [Na]–[Cl] difference, serum creatinine and urine output.

To identify factors associated with AKI and mortality, uni- and multivariate analyses were computed using Cox regression using time-dependent covariates. Variables known to be significantly associated with AKI or mortality were a priori selected to be entered in the models. Serum creatinine, chloremia, vasopressor dose and volume of fluid resuscitation were used as time-dependent covariate. Patients with at least one missing baseline values were excluded from this analysis. Other missing values for time-dependent covariates were imputed by the last and next method [19].

Several chloride parameters were studied, i.e. chloremia within 72 h, hyperchloremia, maximal chloremia, delta chloremia, [Na–Cl] difference. These parameters were separately entered in each model.

To take into account the competing risk of early death, a sensitivity analysis was performed among patients alive at day 3 and different scenario of hyperchloremia imputation were tested in patients dead at day-3 without hyperchloremia. In these patients, hyperchloremia was imputed at H0 alone, H24 alone and at both.

All analyses were performed using R software (R Core Team. R Foundation for Statistical Computing, Vienna Austria, version 3.4.4).

## Results

413 patients of the 434 included could be analysed totalizing 1736 metabolic points recorded. Hyperchloremia was observed in 257 patients (62%) and hyperlactatemia in 294 (71%), *p* = 0.006 (Additional file 1: Figure S1). The cumulative number of patients with hyperchloremia was 111 at H0, 215 at H12, 242 at H24 and 257 at H72. Episodes of hyperlactatemia were more frequent among patients with hyperchloremia, 75% vs. 65%, *p* = 0.04. Hyperchloremia and hyperlactatemia were recorded in the same patient in 192/413 (46%).

### Comparison of patients with and without hyperchloremia

Patients with and without hyperchloremia had similar baseline characteristics (Table 1). Patients with hyperchloremia were more frequently included in the hypertonic saline group and received significantly higher volumes of fluids from H0 to H72: 2.2 (1.1–3.9) vs. 1.4 (0.6–2.3) litres for fluid resuscitation, *p* < 0.001 and 8.2 (5.4–11.4) vs. 5.4 (3.2–8.6) litres for all iv fluids, *p* < 0.001.

**Table 1:** Characteristics and outcomes of patients with and without hyperchloremia

	Not included patients *N* = 21	No hyperchloremia *N* = 156	Hyperchloremia *N* = 257	*P* value^a^
Hypertonic saline group, *n* (%)	13 (62)	45 (29)	156 (61)	*<* *0.001*
Age, year	64 (61–73)	68 (57–77)	69 (59–78)	0.28
Mc Cabe, *n* (%)				0.36
No fatal underlying disease	14 (67)	94 (60)	171 (67)	
Fatal underlying disease at 5 year	6 (29)	44 (17)	65 (25)	
Fatal underlying disease at 1 year	1 (5)	18 (12)	21 (8)	
Sex M, *n* (%)	11 (52)	108 (69)	158 (61)	0.11
Weight, Kg	67 (59–80)	73 (61–85)	70 (61–80)	0.32
Surgical admission, *n* (%)	6 (29)	32 (21)	89 (35)	*0.002*
Cumulative volume of fluids before H0, L	2.5 (2.3–4.0)	2.5 (2.0–3.5)	2.8 (2.0–3.6)	0.28
History of, *n* (%)				
CKD	3 (14)	19 (12)	24 (9)	0.35
Cirrhosis	2 (10)	6 (4)	12 (5)	0.70
Heart failure	0	9 (6)	14 (5)	0.88
Immunosuppression	5 (24)	31 (20)	51 (20)	0.99
Diabetes	4 (19)	33 (21)	49 (19)	0.58
Serum Creatinine at H0, µmol/L^b^	180 (135–241)	133 (79–195)	130 (79–189)	0.21
ARDS at H0, *n* (%)	5 (24)	41 (26)	69 (27)	0.90
SAPS II, points	69 (55–79)	55 (46–63)	56 (48–65)°	0.18
SOFA at H0, points	11 (9–16)	10 (8–12)	10 (8–12)	0.31
Vasopressor dose at H0, µg/Kg/min	0.45 (0.28–1.0)	0.32 (0.20–0.66)	0.44 (0.23–0.80)	*0.04*
Cumulative volume of fluids for resuscitation H0–H72, L	1.4 (0.6–2.2)	1.4 (0.6–2.3)	2.2 (1.1–3.9)	*<0.001*
Outcomes, *n* (%)				
AKI	7 (33)	71 (51)	110 (45)	0.29
Need for RRT^b^	7 (33)	53 (36)	90 (36)	0.98
Make 28	15 (71)	83 (53)	131 (51)	0.66
Day-28 mortality	15 (71)	60 (38)	95 (37)	0.76

MAKE = Major Adverse Kidney Events including either death, need for starting RRT, AKI or persistent AKI

^a^For comparison between patients with and without hyperchloremia

^b^Among patients free of RRT at H0

**Fig. 1:**
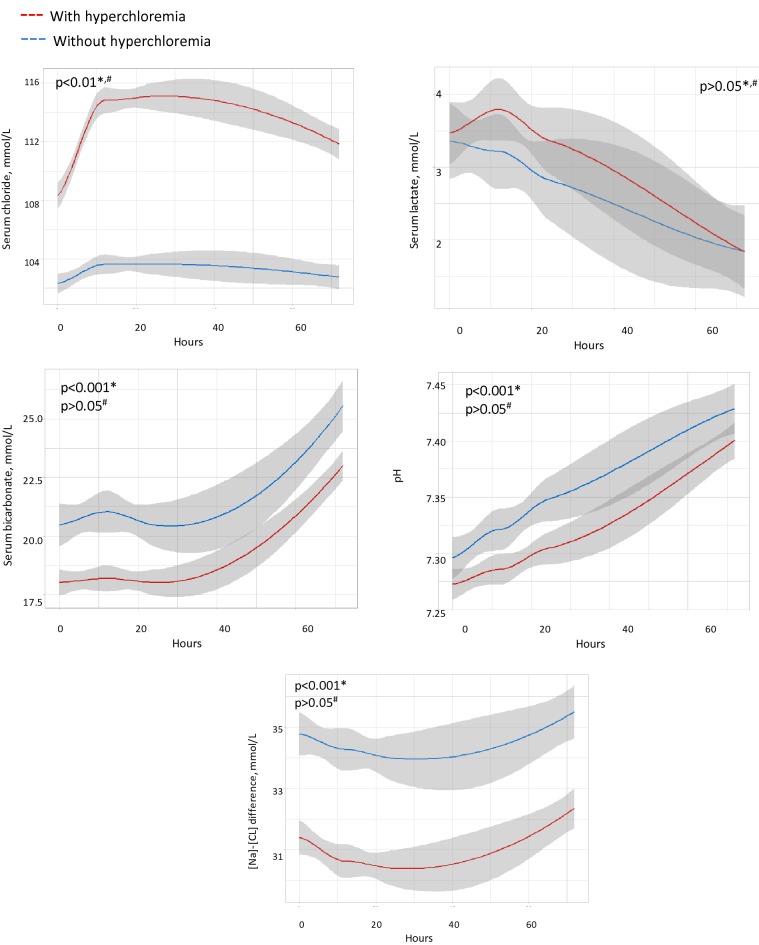
Evolution of metabolic parameters between patient with and without hyperchloremia from H0 to H72. Means and 95% IC are provided. *p value for comparison at H0. ^#^*p* value for comparison of the evolution over time (results of Generalized Linear Mixed Model for pH and Generalized Non-Linear Mixed Model for others)

Figure 1 shows the evolution of metabolic parameters in patients with and without hyperchloremia. Serum chloride was significantly higher all over the time in the group of patients with hyperchloremia. Serum bicarbonate, pH and [Na]–[Cl] difference were significantly lower at H0 among patients with hyperchloremia. The evolution of all parameters (except for chloremia) were similar. Of note, the slopes of acidemia corrections were comparable between patients with and without hyperchloremia (Fig. 1).

No matter the presence of hyperlactatemia, metabolic acidosis was significantly more frequent in patients with hyperchloremia (Additional file 1: Table S2). In patients without hyperlactatemia, acidosis occurred in 60% of patient with hyperchloremia *vs.* 36% without hyperchloremia (*p* < 0.01). In patient with hyperlactatemia, acidosis occurred in 86% of patients with hyperchloremia compared to 64% (*p* < 0.001) in patients without hyperchloremia.

### Impact of hyperchloremia on outcomes

**Fig. 2:**
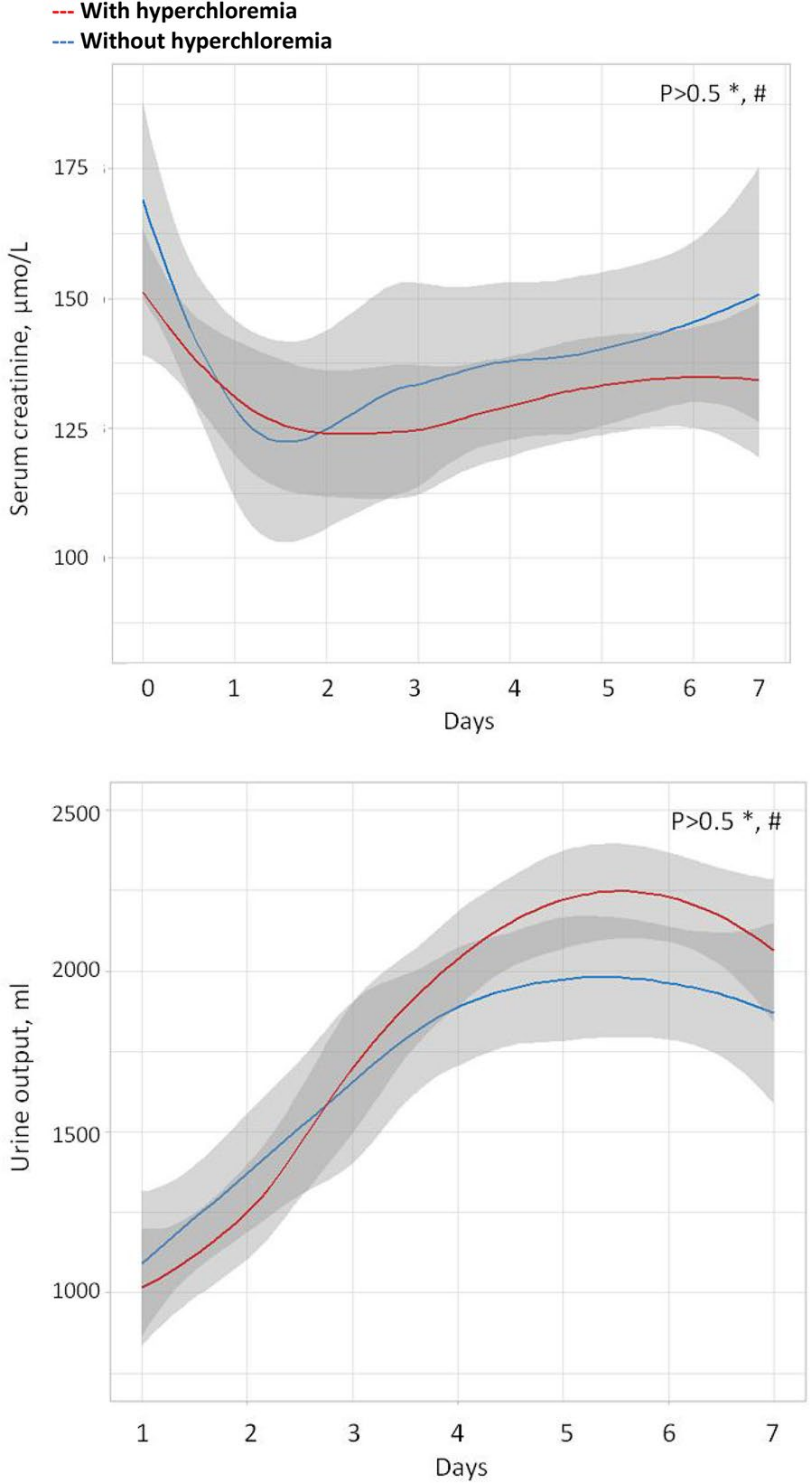
Evolution of renal function between patient with and without hyperchloremia from H0 to day-7 among patient free of RRT at H0. Means and 95% IC are provided. *p value for comparison at H0. ^#^*p* value for comparison of the evolution over time (results of Generalized Non-Linear Mixed Model)

Patients with and without hyperchloremia had similar outcomes regarding AKI (45% vs 51%, *p* = 0.29), need for RRT (36% vs 36%; *p* = 0.98), MAKE-28 (51% vs 53%, *p* = 0.66) (Table 1). The evolution of serum creatinine and urine output were similar between patients with and without hyperchloremia (Fig. 2). The evolution of serum chloride concentration was similar between patients with and without AKI (Additional file 1: Fig S2).

Mortality up to day-28 was 37% vs 38%, *p* = 0.76 in patients with and without hyperchloremia, respectively (Table 1). The evolution of serum chloride concentration was similar between survivors and non survivors (Additional file 1: Fig S2).

In multivariate analysis, the risk of AKI (Table 2) and death (Table 3) was not independently associated with chloride parameters.

**Table 2:** Uni and multivariate analysis for chloride parameters and AKI

	No AKI *N* = 203	AKI *N* = 181	HR 95% CI, *p*	Adjusted HR ^a,b^95% CI, *p*
Hyper chloremia, *n* (%)				
No	69 (34.0%)	71(39.2%)	1 (ref)	1 (ref)
Yes	134 (66.5%)	110 (60.8%)	0.94 [0.67; 1.31], 0.706	1.01 [0.66; 1.52], 0.974
[Chloride], mmol/L	–	–	1.00 [0.98; 1.03], 0.647	1.01 [0.99; 1.03], 0.872
[Chloride] max, mmol/L	112 [107–119]	111 [108 – 117]	1.00 [0.98; 1.02], 0.962	1.00 [0.98; 1.02], 0.792
Delta [Chloride] > 5 mmol/L, *n* (%)				
No	93 (46.5%)	70 (45.2%)	1 (ref)	
Yes	107 (53.5%)	85 (54.8%)	1.04 [0.73;1.48], 0.851	0.94 [0.66; 1.34], 0.724
[Na–Cl] difference, mmol/L	–	–	1.00 [0.99; 1.02], 0.852	1.00 [0.98; 1.03], 0.914
[Na–Cl] minimal difference, mmol/L	30 [28–33]	30 [27–33]	0.99 [0.96; 1.04], 0.834	1.01 [0.96; 1.05], 0.830

^a^Adjusted for SAPS, surgical admission, weigh, chronic kidney disease, diabetes, SOFA and ARDS at H0 and for the following time-dependent variables: serum creatinine, vasopressor dose, volume of fluid resuscitation

^b^Adjusted models were developed in the 340 patients free of RRT at inclusion and without missing data

**Table 3:** Uni and multivariate analysis for chloride parameters and mortality

	Survivors *N* = 258	Non survivors *N* = 155	HR 95% CI, *p*	Adjusted HR^a, #^ 95% CI, *p*
Hyper chloremia, *n* (%)				
No	96 (37.2%)	60 (38.7%)		1 (ref)
Yes	162 (62.8%)	95 (61.3%)	0.87 [0.61;1.27], 0.509	0.88 [0.96; 1.00], 0.061
[Chloride], mmol/l	–	–	0.98 [0.96; 1.00], 0.193	0.99 [0.97; 1.01], 0.175
[Chloride] max, mmol/L	112 [107–118]	111 [106.5–117]	0.99 [0.97;1.01], 0.363	0.98 [0.96; 0;99], 0.043
Delta [Chloride] > 5 mmol/L, *n*				
No	103 (44.8%)	67 (48.2%)	1 (ref)	1 (ref)
Yes	127 (55.2%)	72 (51.8%)	0.88 [0.61; 1.26], 0.492	0.72 [0.49; 1.04], 0.081
[Na–Cl] difference, mmol/L	–	–	1.00 [0.93; 1.08], 0.942	1.00 [0.93; 1.09], 0.924
[Na–Cl] minimal difference, mmol/L	30 [28–32]	30 [27–34]	1.02 [0.98; 1.07], 0.285	1.03 [0.98; 1.08], 0.286

^a^Adjusted for hyperoxia group, age, MacCabe, SAPS, SOFA, Hyperlactatemia, Serum creatinine and ARDS at H0 and for the following time-dependent variable: Serum creatinine, vasopressor dose, Serum lactate and volume of fluid resuscitation

^#^Adjusted models were developed in the 352 patients without missing data

### Sensitivity analysis

Among the 73 patients who died early, only 29 were not exposed to hyperchloremia. Sensitivity analysis performed in patients still alive at days 3 or with the imputation of hyperchloremia is reported in Additional file 1: Table S3. Sensitivity analysis of AKI and death did not modify the main results. Nonetheless, we observed a trend toward an increased risk of AKI when hyperchloremia is imputed to the 29 patients at H24, but not at H0.

## Discussion

We report the first analysis of the potential role of chloremia on the outcome of patients with septic shock. Our results show that hyperchloremia was frequent in patients with septic shock resuscitated with chloride rich crystalloids, however, significantly less frequent than hyperlactatemia. Independently of the presence of hyperlactatemia, hyperchloremia was associated with more frequent metabolic acidosis and more pronounced acidemia. Nevertheless, resuscitation using chloride rich fluids did not worsen shock-related metabolic acidosis nor impeded its correction, even in the presence of hyperchloremia. Hyperchloremia was not associated with an increased risk for AKI or death.

Strong associations between high chloride levels and worse outcome, AKI or death, have been reported; however, in various cohort studies discrepant results exist [12, 13, 20, 21]. Our findings are consistent with several negative retrospective cohorts assessing the role of hyperchloremia in ICU patients. In a single centre study enrolling 1045 patients with sepsis, 29% of them developing hyperchloremia, Yessayan et al. [22] did not show an association between hyperchloremia nor delta chloremia (as defined by increase > 5 mmol/L) and AKI. In a multicentre study including patients with severe acidemia, 54% of them with septic shock, the pH value and lactatemia were significantly associated with increased mortality, while chloremia was not [23]. Similarly, in a general population of ICU patients, Gunnerson et al. [24] found that lactate was a strong and independent predictor of mortality contrary to chloremia. A moderate increase in serum chloride of 5 mmol/L, even in patients with chloremia in normal range, has been shown to impair renal function and worsen survival in septic patients [12, 13]. We, therefore, tested different chloride parameters (i.e., hyperchloremia, chloremia as a time-dependent variable, maximal concentration, increase in concentration > 5 mmol/L); none of them was significantly associated with neither AKI or mortality. The negative results of our study deserve discussion of the underlying hypothesis. We assessed the role of chloremia in the most severe population reported so far. On the one hand, severe patients who receive large volumes of fluids are more exposed to the risk of hyperchloremia and its related adverse effects, on the other hand, stronger factors determining renal function and survival could hide the specific effect of chloremia. In patients with shock, the benefit of fluids on shock reversal could outweigh the risks of hyperchloremia. In our study, despite the occurrence of hyperchloremia, fluid resuscitation did not worsen acidemia, which is in contrast to data reported in surgical patients with normal pH at baseline [25]. Of note, in cohort studies reporting a negative impact of chloride load, the volume of fluid administered (i.e. around 5 litres within 24 or 48 h) was usually higher than in our study, despite a lower severity of patients studied [26–28].

We assessed the impact of hyperchloremia in the context of hypertonic fluid resuscitation. Occurrence of hyperchloremia was, therefore, driven by hypernatremia which per se might have a different impact on the outcome. However, the reduced difference between sodium and chloride concentrations indicating excessive chloride load was not associated with worse outcome. In trauma patients, hypertonic fluid resuscitation induced a huge, but transitory, increase in chloremia [29]. Compared to isotonic crystalloids, hypertonic solutions have never been identified as a significant risk for inducing AKI under these conditions [30].

The mechanism by which hyperchloremia would induce AKI remains highly speculative, particularly in the context of and the presence of markedly altered vasomotion. Comparing NaCl solutions of different chloride concentrations, animal studies have suggested that infusing a chloride-rich solution, directly into the renal artery, critically determined changes in renal blood flow due to intra-renal vasoconstriction [31, 32]. This finding was not confirmed in a more relevant animal model of sepsis resuscitated with large amounts of crystalloids [33]. Compared to a balanced solution, normal saline induced hyperchloremia and acidosis; however, no difference was found for renal hemodynamics and function [33]. Moreover, decreased renal blood flow velocity and cortical tissue perfusion have been reported in healthy volunteers after infusion of 2L of saline [34]. Finally, ICU patients in whom renal perfusion was assessed by renal Doppler, no relation was found between chloremia and resistive index [35].

Our study has several strengths: (i) the study design combines a number of features that reduce the risk of bias, (ii) metabolic parameters were prospectively recorded at precise time points with a concomitant record of serum lactate and chloride allowing precise adjustment, (iii) the choice of the fluids, the metabolic parameters to be recorded and the renal monitoring were imposed by the study design. Hence, in contrast to previous retrospective cohort studies based on administrative funding databases, biases related to fluid selection and metabolic dosage determined by patient characteristic and physician choices were ruled out. Such biases are present in the “SMART” trial in which the type of balanced solution, metabolic and renal function monitoring were left at the discretion of investigators [4].

Our study also has limitations that deserve attention. It is a post hoc analysis and the number of patients, hence, is quite limited. Nevertheless, it is noteworthy that no trend of worse evolution was observed among patient with hyperchloremia. Of note, we observed a trend toward higher risk of AKI in the sensitivity analysis when hyperchloremia was imputed to the early dying patient at H24 but not at H0 (Additional file 1: Table S3). Crystalloids used were only chloride rich with a high proportion of patient developing hyperchloremia. However, a sufficient number of patients remained free of hyperchloremia. The rules applied in the study for stopping hypertonic saline when hypernatremia occurred might have minimized the risk and the intensity of hyperchloremia. However, hyperchloremia over beyond 120 mmol/l was observed in 25% of the patients (Additional file 1: Table S2).

Our study did not assess the potential beneficial effect of using balanced crystalloid. The resolution of metabolic acidosis could have been more rapid using balanced solutions. The impact of the rapidity of acidemia correction on the patients’ evolution, however, remains unknown. Our results merely suggest that the potential beneficial effects of balanced solution in septic shock patients are might be unrelated to the serum chloride concentration. The definition of AKI was based on serum creatinine level at inclusion in the “HYPER2S” study. Using baseline serum creatinine before septic shock occurrence may have allowed detection and exclusion of patients with AKI occurrence before exposure and may have resulted in different finding. We used baseline creatinine at H0, i.e. just before exposure, because otherwise patients developing AKI before the start of sepsis start and exposure to chloride-rich solutions (H0) would have been wrongly classified as AKI-related to hyperchloremia. Considering the design of our study and the large confidence intervals of associations, our results do not rule out a negative association between chloride parameters and outcomes in patients with septic shock. Our results need to be confirmed by a larger study specifically designed to address this question in patients with septic shock of similar severity.

In conclusion, patients with septic shock resuscitated with chloride rich crystalloids are frequently exposed to hyperchloremia. However, the impact of hyperchloremia on outcomes might be negligible compared to the inherent risks from acute disease. Further studies are required to identify the specific role of chloride disturbances and use of balanced crystalloids on the outcome of septic shock patients.

## **Supplementary information**

**Additional file 1: Fig S1.** Patients classification according to the presence of hyperchloremia > 110 mmol/L and/or hyperlactatemia > 2 mmol/L. **Fig S2.** Evolution of serum chloride concentration between patients with and without AKI (panel A) and between survivors and non survivors (panel B) from H0 to H72. Indicated values correspond to mean and 95% IC. For AKI, analysis was done among patient free of RRT at H0. *p value for comparison at H0. #*p* value for comparison of the evolution over time (results of the Generalized Non-Linear Mixed Models)**. Table S1.** Definitions of metabolic parameters and of fluids administered. **Tables S2.** Evolution of metabolic parameters from H0 to H72 in patients with and without hyperchloremia stratified by hyperlactatemia**. Table S3.** Results of the sensitivity analysis.

## Data Availability

The datasets used and/or analysed during the current study are available from the corresponding author on reasonable request

## Abbreviations

AKI: acute kidney injury
Cl: chloride
ICU: intensive care unit
MAKE: major adverse kidney events
NaCl: sodium chloride
RCT: randomized controlled trial

## References

[1] Rhodes A, Evans LE, Alhazzani W (2017). Surviving sepsis campaign: international guidelines for management of sepsis and septic shock: 2016. Intensive Care Med.

[2] Hammond NE, Taylor C, Finfer S (2017). Patterns of intravenous fluid resuscitation use in adult intensive care patients between 2007 and 2014: an international cross-sectional study. PLoS ONE.

[3] Pfortmueller CA, Uehlinger D, von Haehling S (2018). Serum chloride levels in critical illness-the hidden story. Intensive care Med Exp.

[4] Semler MW, Self WH, Wanderer JP (2018). Balanced crystalloids versus saline in critically ill adults. N Engl J Med.

[5] Young P, Bailey M, Beasley R (2015). Effect of a buffered crystalloid solution vs saline on acute kidney injury among patients in the intensive care unit: the SPLIT randomized clinical trial. JAMA.

[6] Semler MW, Wanderer JP, Ehrenfeld JM (2017). Balanced crystalloids versus saline in the intensive care unit. The SALT randomized trial. Am J Respir Crit Care Med.

[7] Xue M, Zhang X, Liu F (2019). Effects of chloride content of intravenous crystalloid solutions in critically ill adult patients: a meta-analysis with trial sequential analysis of randomized trials. Ann Intensive Care.

[8] Semler MW, Kellum JA (2018). Balanced crystalloid solutions. Am J Respir Crit Care Med.

[9] Kellum JA, Song M, Li J (2004). Lactic and hydrochloric acids induce different patterns of inflammatory response in LPS-stimulated RAW 264.7 cells. Am J Physiol Regul Integr Comp Physiol.

[10] Asfar P, Schortgen F, Boisrame-Helms J (2017). Hyperoxia and hypertonic saline in patients with septic shock (HYPERS2S): a two-by-two factorial, multicentre, randomised, clinical trial. Lancet Respir Med.

[11] McCluskey SA, Karkouti K, Wijeysundera D (2013). Hyperchloremia after noncardiac surgery is independently associated with increased morbidity and mortality: a propensity-matched cohort study. Anesth Analg.

[12] Neyra JA, Canepa-Escaro F, Li X (2015). Association of hyperchloremia with hospital mortality in critically ill septic patients. Crit Care Med.

[13] Suetrong B, Pisitsak C, Boyd JH (2016). Hyperchloremia and moderate increase in serum chloride are associated with acute kidney injury in severe sepsis and septic shock patients. Crit Care.

[14] Shaw AD, Schermer CR, Lobo DN (2015). Impact of intravenous fluid composition on outcomes in patients with systemic inflammatory response syndrome. Crit Care.

[15] Singer M, Deutschman CS, Seymour CW (2016). The third international consensus definitions for sepsis and septic shock (sepsis-3). JAMA.

[16] Acute Kidney Injury work group (2012). Improving global outcomes (KDIGO), KDIGO clinical practice guideline for acute kidney injury. Kidney Inter Suppl.

[17] Palevsky PM, Molitoris BA, Okusa MD (2012). Design of clinical trials in acute kidney injury: report from an NIDDK workshop on trial methodology. Clin J Am Soc Nephrol.

[18] Demiselle J, Wepler M, Hartmann C (2018). Hyperoxia toxicity in septic shock patients according to the Sepsis-3 criteria: a post hoc analysis of the HYPER2S trial. Ann Intensive Care.

[19] Engels JM, Diehr P (2003). Imputation of missing longitudinal data: a comparison of methods. J Clin Epidemiol.

[20] Marttinen M, Wilkman E, Petaja L (2016). Association of plasma chloride values with acute kidney injury in the critically ill—a prospective observational study. Acta Anaesthesiol Scand.

[21] Shaw AD, Raghunathan K, Peyerl FW (2014). Association between intravenous chloride load during resuscitation and in-hospital mortality among patients with SIRS. Intensive Care Med.

[22] Yessayan L, Neyra JA, Canepa-Escaro F (2017). Effect of hyperchloremia on acute kidney injury in critically ill septic patients: a retrospective cohort study. BMC Nephrol.

[23] Jung B, Rimmele T, Le Goff C (2011). Severe metabolic or mixed acidemia on intensive care unit admission: incidence, prognosis and administration of buffer therapy. A prospective, multiple-center study. Crit Care.

[24] Gunnerson KJ, Saul M, He S (2006). Lactate versus non-lactate metabolic acidosis: a retrospective outcome evaluation of critically ill patients. Crit Care.

[25] Scheingraber S, Rehm M, Sehmisch C (1999). Rapid saline infusion produces hyperchloremic acidosis in patients undergoing gynecologic surgery. Anesthesiology.

[26] Raghunathan K, Shaw A, Nathanson B (2014). Association between the choice of IV crystalloid and in-hospital mortality among critically ill adults with sepsis*. Crit Care Med.

[27] Sen A, Keener CM, Sileanu FE (2017). Chloride content of fluids used for large-volume resuscitation is associated with reduced survival. Crit Care Med.

[28] Zampieri FG, Ranzani OT, Azevedo LC (2016). Lactated ringer is associated with reduced mortality and less acute kidney injury in critically ill patients: a retrospective cohort analysis. Crit Care Med.

[29] Cooper DJ, Myles PS, McDermott FT (2004). Prehospital hypertonic saline resuscitation of patients with hypotension and severe traumatic brain injury: a randomized controlled trial. JAMA.

[30] Wu MC, Liao TY, Lee EM (2017). Administration of hypertonic solutions for hemorrhagic shock: a systematic review and meta-analysis of clinical trials. Anesth Analg.

[31] Quilley CP, Lin YS (1993). McGiff JC Chloride anion concentration as a determinant of renal vascular responsiveness to vasoconstrictor agents. Br J Pharmacol.

[32] Wilcox CS (1983). Regulation of renal blood flow by plasma chloride. J Clin Invest.

[33] Olivier PY, Beloncle F, Seegers V (2017). Assessment of renal hemodynamic toxicity of fluid challenge with 0.9% NaCl compared to balanced crystalloid (PlasmaLyte((R))) in a rat model with severe sepsis. Ann Intensive Care.

[34] Chowdhury AH, Cox EF, Francis ST (2012). A randomized, controlled, double-blind crossover study on the effects of 2-L infusions of 0.9% saline and plasma-lyte(R) 148 on renal blood flow velocity and renal cortical tissue perfusion in healthy volunteers. Ann Surg.

[35] Oliveira RAG, Mendes PV, Park M (2019). Factors associated with renal Doppler resistive index in critically ill patients: a prospective cohort study. Ann Intensive Care.

