# Supplementary material for: Hyperchloremia is not associated with AKI or death in septic shock patients: results of a post hoc analysis of the “HYPER2S” trial
Source: Ann Intensive Care. 2019 Aug 22;9:95. doi: 10.1186/s13613-019-0570-3 (PMC6706496; doi:10.1186/s13613-019-0570-3)
Supplement: Supplementary file 1 — Additional file 1: Fig S1. Patients classification according to the presence of hyperchloremia > 110 mmol/L and/or hyperlactatemia > 2 mmol/L. Fig S2. Evolution of serum chloride concentration between patients with and without AKI (panel A) and between survivors and non survivors (panel B) from H0 to H72. Indicated values correspond to mean and 95% IC. For AKI, analysis was done among patient free of RRT at H0. *p value for comparison at H0. #p value for comparison of the evolution over time (results of the Generalized Non-Linear Mixed Models). Table S1. Definitions of metabolic parameters and of fluids administered. Tables S2. Evolution of metabolic parameters from H0 to H72 in patients with and without hyperchloremia stratified by hyperlactatemia. Table S3. Results of the sensitivity analysis. [file 13613_2019_570_MOESM1_ESM.doc]

**Additional file**

**Hyperchloremia is not associated with AKI and death in septic shock patients: results of a post hoc analysis of the “HYPER2S” trial**

MorganeCommereuc^1^, Camille Nevoret^2, 3, 4^, Peter Radermacher^5^, Sandrine Katsahian^2, 3, 4^, Pierre Asfar^6^, Frédérique Schortgen^1, 7^on behalf of the HYPER2S investigators

1. Réanimation adulte et surveillance continue, Centre hospitalier Intercommunal de Créteil 94000, Créteil-France
2. INSERM, UMR_S 1138, Université Paris Descartes, Sorbonne Universités, UPMC Université Paris 06, Centre de Recherche des Cordeliers, Paris, France.
3. Assistance Publique - Hôpitaux de Paris, Hôpital Européen Georges-Pompidou, Unité d'Épidémiologie et de Recherche Clinique, Paris, France.
4. INSERM, Centre d'Investigation Clinique 1418, Module Épidémiologie Clinique, Paris, France.
5. Institut fürAnästhesiologische Pathophysiologie undVerfahrensentwicklung, Universitätsklinikum, Ulm, Germany (Universitätsklinikum Ulm Helmholtzstr. 8/1 89081 Ulm, GERMANY)
6. Département de Médecine Intensive-Réanimation et Médecine Hyperbare, CHU d’Angers, France
7. INSERM U955 Equipe 13, Faculté de Médecine, 94010, Créteil France

**Correspondingauthor:**

Frédérique Schortgen

Service de réanimation-USC adulte

Centre hospitalier Intercommunal de Créteil

94000 Créteil France

[Frederique.schortgen@chicreteil.fr](mailto:Frederique.schortgen@chicreteil.fr)

Tel: 00 33 1 45 17 56 83

ORCID number: 0000-0003-1756-7508

**Figure S1:** Patients classification according to the presence of hyperchloremia > 110 mmol/L and/or hyperlactatemia > 2 mmol/L

**Figure S2: Evolution of serum chloride concentration between patients with and without AKI (panel A) and between survivors and non survivors (panel B) from H0 to H72**

Indicated values correspond to mean and 95% IC

For AKI, analysis was done among patient free of RRT at H0

* p value for comparison at H0

# p value for comparison of the evolution over time (results of the Generalized Non Linear Mixed Models)

**Table S1:** Definitions of metabolic parameters and of fluids administered

**Tables S2**: Evolution of metabolic parameters from H0 to H72 in patients with and without hyperchloremia stratified by hyperlactatemia

**Table S3: Results of the sensitivity analysis**

**Investigators of the “HYPER 2S” trial**

**Figure S1: Patients classification according to the presence of hyperchloremia > 110 mmol/l and/or hyperlactatemia> 2 mmol/l**

413

**Figure S2: evolution of serum chloride concentration between patients with and without AKI (panel A) and between survivors and non survivors (panel B) from H0 to H72**

**Panel A**

**
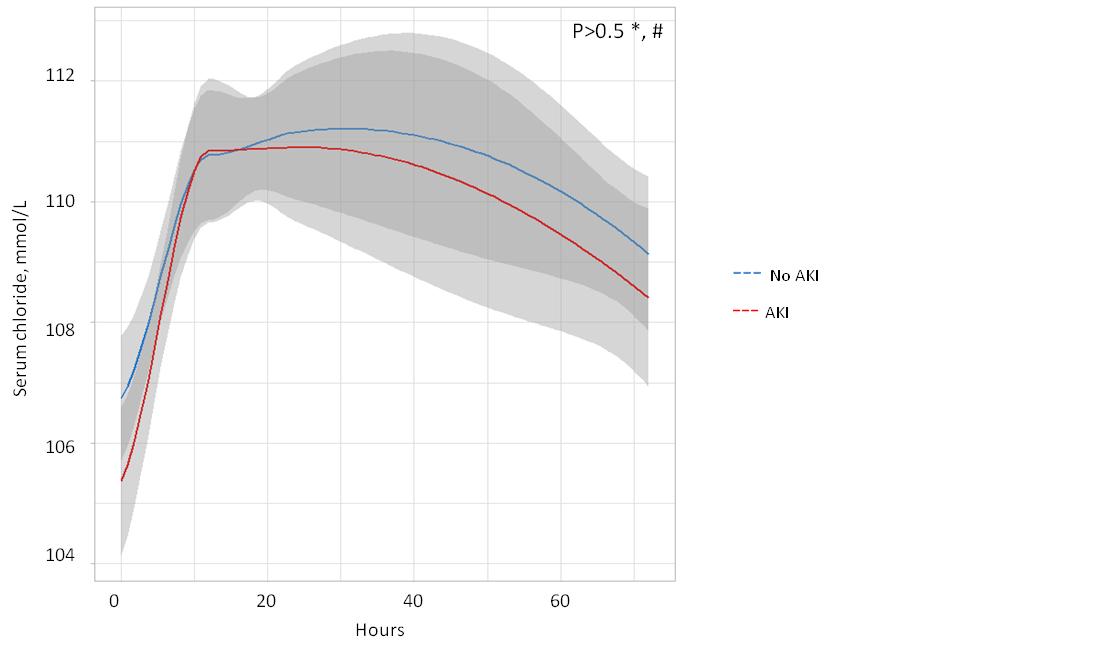
**

**Panel B**

**
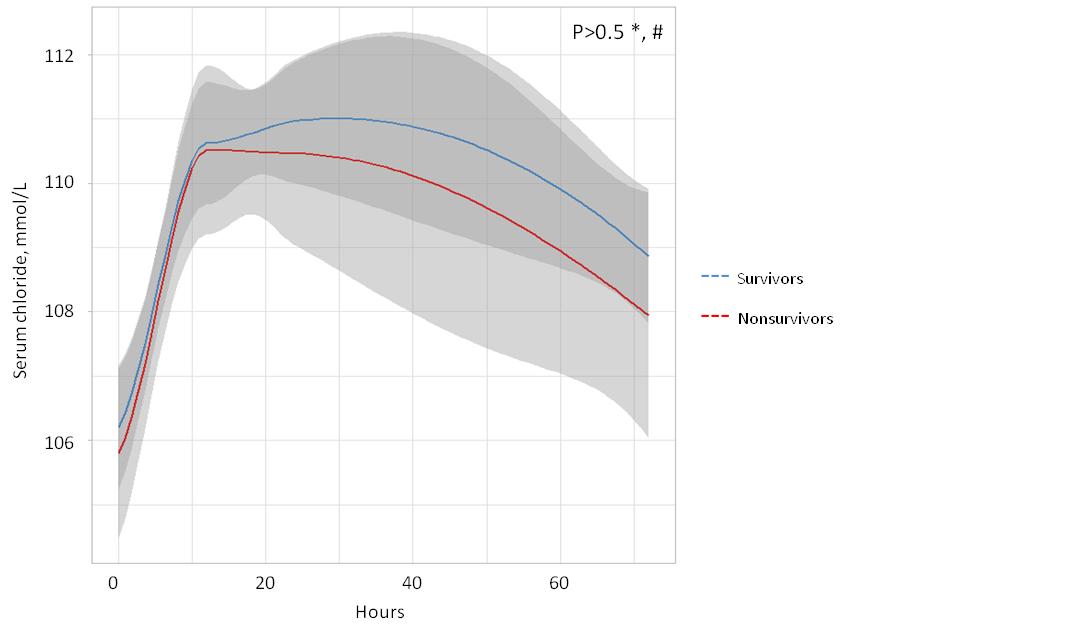
**

**Table S1:** definitions of metabolic parameters and of fluids administered

| **Hyperlactatemia** | **[Lactate]>2 mmol/L** |
| --- | --- |
| **Hyperchloremia** | **[Cl]>110 mmol/L** |
| **Acidemia** | **pH<7.35** |
| **Metabolic acidosis** | **pH<7.35 + [HCO_3_^-^] <22 mmol/L** |
| **MaximalChloremia** | **Maximal value of [Cl] among H0 H12 H24 H72** |
| **Delta Chloremia** | **Max [Cl] –[Cl]at H0** |
| **MeanChloremia** | **∑[Cl]H0 H12 H24 H72/n values** |
| **Mean pH** | **∑ pH H0 H12 H24 H72/n values** |
| **Mean lactate** | **∑[lact] H0 H12 H24 H72/n values** |
| **Mean PaCO2** | **∑ PaCO_2_ H0 H12 H24 H72/n values** |
| **Meanbicarbonate** | **∑[HCO_3_^-^] H0 H12 H24 H72/n values** |
| **Na-Cl difference** | **[Na]-[Cl] mmol/L** |
| **Mean Na-CL difference** | **∑ [Na]-[Cl] H0 H12 H24 H72/n values** |
| **Minimal Na-Cldifference** | **Minimal value of [Na]-[Cl] mmol/Lamong H0 H12 H24 H72** |
| **Volume of fluids for resuscitation** | **∑ Volume of NaCl 3% and NaCl 0.9% from H0 to H72** |
| **Volume of all IV fluids** | **∑ Volume of all fluids administered intravenously (excepting nutrition) from H0 to H72** |

**Tables S2**: Evolution of metabolic parameters from H0 to H72

|  | **Without hyperlactatemia** | |  | | **With hyperlactatemia** | | | |  | |
| --- | --- | --- | --- | --- | --- | --- | --- | --- | --- | --- |
|  | **Hyperchloremia**  **NO**  **n=54** | **Hyperchloremia**  **YES**  **n=65** | | **p** | | **Hyperchloremia**  **NO**  **n=102** | | **Hyperchloremia**  **YES**  **n=192** | | **p** |
| **[Lactate] max, mmol/L** | 1.5 (1.3-1.7) | 1.5 (1.4-1.8) | 0.16 | | 3.8 (2.8-7.3) | | 3.8 (2.9-7.7) | | 0.46 | |
| **[Lactate] mean, mmol/L** | 1.2 (1.1-1.4) | 1.3 (1.1-1.5) | 0.16 | | 2.8 (2.0-4.7) | | 2.8 (2.0-4.9) | | 0.82 | |
| **[Chloride] max, mmol/L** | 106 (103-108) | 115 (114-120) | **<0.001** | | 106 (104-108) | | 116 (112-123) | | **<0.001** | |
| **[Chloride] mean, mmol/L** | 104 (101-105) | 112 (110-116) | **<0.001** | | 104 (102-106) | | 112 (109-116) | | **<0.001** | |
| **[Na-Cl] mean, mmol/l** | 34 (32-37) | 31 (29-33) | **<0.001** | | 35 (32-37) | | 31 (29-33) | | **<0.001** | |
| **Mean PaCO2, mmHg** | 41 (34-49) | 40 (37-44) | 0.73 | | 39 (33-45) | | 37 (33-40) | | **0.01** | |
| **[HCO3] mean, mmol/l** | 23 (20-28) | 21 (19-23) | **0.02** | | 21 (18-23) | | 19 (16-20) | | **<0.001** | |
| **Mean pH** | 7.38 (7.32-7.43) | 7.35 (7.31-7.38) | **0.02** | | 7.34 (7.27-7.40) | | 7.31 (7.25-7.36) | | **0.006** | |
| **Patients with >1 episode of metabolic acidosis, n (%)** | 17 (31) | 39 (60) | **0.002** | | 65 (64) | | 165 (86) | | **<0.001** | |
| **Number of episodes of metabolic acidosis per patient** | 0 (0-1) | 1 (0-2) | **0.006** | | 1 (0-2) | | 2 (1-3) | | **<0.001** | |

**Table S3: Results of the sensitivity analysis**

|  | **Mortality** | **AKI** |
| --- | --- | --- |
|  | **Adjusted HR 95% CI** | **Adjusted HR 95% CI** |
| **All patients** |  |  |
| Hyperchloremia, n (%) |  |  |
| No | 1 | 1 |
| Yes | 0.62 [0.36; 1.06], 0.08 | 1.01 [0.66 ; 1.52], 0.974 |
|  |  |  |
| **Day-3 survivors only** |  |  |
| Hyper Chloremia, n (%) |  |  |
| No | 1 | 1 |
| Yes | 1.10 [0.65, 1.86], 0.717 | 0.91 [0.53, 1.58], 0.74 |
|  |  |  |
| **Hyperchloremia imputed at H0** |  |  |
| Hyper Chloremia, n (%) |  |  |
| No | 1 | 1 |
| Yes | 0.68 [0.40, 1.16], 0.158 | 1.01 [0.67, 1.52], 0.974 |
|  |  |  |
| **Hyperchloremia imputed at H0 and H24** |  |  |
| Hyper Chloremia, n (%) |  |  |
| No | 1 | 1 |
| Yes | 1.22 [0.72, 2.09], 0.358 | 1.52 [0.99, 2.32], 0.052 |
|  |  |  |
| **Hyperchloremia imputed at H24** |  |  |
| Hyper Chloremia, n (%) |  |  |
| No | 1 | 1 |
| Yes | 1.12 [0.65, 1.91], 0.689 | 1.52 [1.00, 2.31], 0.051 |

**Investigators of the HYPER2S trial**

Angers University Hospital, department of medical intensive care medicine and hyperbaric medicine (Dr KOUATCHET Achille, Dr SOUDAY Vincent, Dr PIERROT Marc, Pr LEROLLE Nicolas, Dr MORTAZA Satar, Dr BOUVIER Guillaume, Dr DONZEAU Alexis, Dr CHUDEAU Nicolas, Dr HUBERT Noémie, Dr CONTE Mathieu, Mr OLIVIER Clément, Mr HOGUIN Maxime, Melle SCHWANKA Justine, Mme MASSON Laure, Melle GUYON Marion), NouvelHôpital Civil, University of Strasbourg (Dr DELABRANCHE Xavier, Dr KUMMERLEN Christine, Dr RABOUEL Yannïck), Cochin University Hospital, Paris (Pr MIRA Jean-Paul, Dr DAVIAUD Fabrice, Dr MARIN Nathalie), Georges Pompidou European Hospital, Paris (Pr DIEHL Jean-Luc, Dr VENOT Marion, Dr LORTAT-JACOB Brice, Dr NOVARA Ana), Lariboisière University Hospital, Paris (Pr BAUD Frédéric, Dr CHESINSKI Anthony, Dr DEYE Nicolas, Dr MALISSIN Isabelle, Dr VODOVAR Dominique, Dr EKHERIAN Jean-Michel, Dr SEBOURCE-GOGUEL Jean), André Mignot University Hospital, Le Chesnay (Pr BEDOS Jean-Pierre, Dr MERCERON Sybille), Le Mans Hospital (Dr TAYORO Jérôme, Dr SACI Slimane, Dr VIVIER Dominique, Dr BILDEA Adriana, Dr TIROT Patrice, Dr BRODEUR Sarah, Dr CALLAHAN Jean-Christophe, Dr Le MOAL, Dr Christophe GUITTON), Bicêtre University Hospital, Le Kremlin-Bicêtre (Dr AMILIEN Virginie, Dr GUERIN Laurent, Dr DRES Martin, Dr MAGALHAES Eric, Dr OSMAN David), Angers University Hospital, department of surgical intensive care medicine (Dr GERGAUD Soizic, Dr GAILLARD Thomas, Dr FESARD Philippe, Mme FRANCOIS Caroline ), La Roche sur Yon Hospital (Dr REIGNIER Jean, Dr LACHERADE Jean-Claude, Dr LEBERT Christine, Dr MARTIN-LEFEVRE Laurent, Dr VINATIER Isabelle, Dr FIANCETTE Maud, Dr LASCARROU Jean-Baptiste, Dr YEHIA Aihem, Dr BACHOUMAS Konstantinos), Avicenne University Hospital, Bobigny (Pr COHEN Yves), Saint Brieuc Hospital (Dr BARBAROT Nicolas, Dr COURTE Anne, Dr BOUSSER Jérôme, Dr MARJOT-ZIMBACCA France, Dr GODARD Aurélie, Dr GUIVARCH Gilbert, Dr DEBARRE Matthieu, Dr SALAH Amor), Nantes University Hospital (Pr VILLERS Daniel, Dr BRETONNIERE Daniel, Dr NICOLET Laurent, Dr ZAMBOU Olivier, Dr BRULE Noëlle, Dr LORBER Julien, Dr AZAIS Marie-Ange), Hautepierre University Hospital, Strasbourg (Dr HERBRECHT Jean-Etienne, Pr SCHNEIDER Francis), Chalon sur Saône Hospital (Dr COMTE Thierry, Dr POUSSANT Thomas, Dr LHOUMEAU Adrien, Dr DELORME Martial, Dr ARTMANN Alexandre) Louis Mourier University Hospital, Colombes (Dr GAUDRY Stéphane, Dr JEAN-BAPTISTE Sylvain, Pr RICARD Jean-Damien), Foch Hospital, Suresnes ( Dr CERF Charles, Dr TREBBIA Grégoire), Saint Philibert University Hospital, Lomme (Dr FOURDIN Caroline, Dr MINACORI Franck, Dr LEDEIN Marie), Poitiers University Hospital (Dr CABASSON Séverin, Pr ROBERT René), Dieppe Hospital (Dr DECLERCQ Pierre-Louis), Rouen University Hospital (Dr GRANGE Steven).
